# Refining the *Maritime Foundations of Andean Civilization*: How Plant Fiber Technology Drove Social Complexity During the Preceramic Period

**DOI:** 10.1007/s10816-017-9341-3

**Published:** 2017-06-29

**Authors:** David Beresford-Jones, Alexander Pullen, George Chauca, Lauren Cadwallader, Maria García, Isabel Salvatierra, Oliver Whaley, Víctor Vásquez, Susana Arce, Kevin Lane, Charles French

**Affiliations:** 1 McDonald Institute for Archaeological Research, University of Cambridge, Downing St., Cambridge, CB2 3ER UK; 2 Pre-Construct Archaeology, The Granary, Rectory Farm, Pampisford, Cambridgeshire CB22 3EN UK; 3 Escuela Profesional de Arqueología, Universidad Nacional Mayor de San Marcos, Av. Universitaria s/n., Lima, Peru; 4 Instituto Colombiano de Antropología e Historia, Calle 12 Numero 2 – 41, Bogotá, DC Colombia; 5 Royal Botanical Gardens Kew, Surrey, Richmond TW9 3AE UK; 6 ArqueoBios, Apartado Postal, 595 Trujillo, Peru; 7 Museo Regional de Ica, Ministerio de Cultura, Ica, Av. Ayabaca s.n.o; cuadra 8, urb. San Isidro, Ica Peru; 8 Instituto de Arqueología, Universidad de Buenos Aires, 25 de Mayo 221 piso 3, 1002 Buenos Aires, Argentina

**Keywords:** Maritime Foundations of Andean Civilization, Preceramic Period, South coast Peru, Complex society, Broad Spectrum Revolution, Cotton, Plant bast fibers, Fishing nets

## Abstract

Moseley’s (1975) *Maritime Foundations of Andean Civilization* hypothesis challenges, in one of humanity’s few pristine hearths of civilization, the axiom that agriculture is necessary for the rise of complex societies. We revisit that hypothesis by setting new findings from La Yerba II (7571–6674 Cal bp) and III (6485–5893 Cal bp), Río Ica estuary, alongside the wider archaeological record for the end of the Middle Preceramic Period on the Peruvian coast. The La Yerba record evinces increasing population, sedentism, and “Broad Spectrum Revolution” features, including early horticulture of *Phaseolus* and *Canavalia* beans. Yet unlike further north, these changes failed to presage the florescence of monumental civilization during the subsequent Late Preceramic Period. Instead, the south coast saw a profound “archaeological silence.” These contrasting trajectories had little to do with any relative differences in *marine* resources, but rather to restrictions on the *terrestrial* resources that determined a society’s capacity to intensify exploitation of those marine resources. We explain this apparent miscarriage of the *Maritime Foundations of Andean Civilization* (MFAC) hypothesis on the south coast of Peru by proposing more explicit links than hitherto, between the detailed technological aspects of marine exploitation using plant fibers to make fishing nets and the emergence of social complexity on the coast of Peru. Rather than because of any significant advantages in *quality*, it was the potential for increased *quantities* of production, inherent in the shift from gathered wild *Asclepias* bast fibers to cultivated cotton, that inadvertently precipitated revolutionary social change. Thereby refined, the MFAC hypothesis duly emerges more persuasive than ever.

## Introduction

The seas off the coast of Peru are the world’s richest fishery. Nutrients in cold upwellings from the ocean abyss here support a prodigious marine food chain. Today, the annual catch of Peruvian anchovy (*Engraulis ringens*) averages around 6 million tons: the greatest harvest of any fish species in the wild (FAO 2014). Over 40 years ago now, Michael Moseley (1975) first synthesized ideas latent in the thinking of Frédéric Engel, Rosa Fung, Edward Lanning, and others, to promulgate the idea that civilization in the Andean region had first arisen based upon the exploitation of such marine resources. This *Maritime Foundations of Andean Civilization* (MFAC) hypothesis argues, in sum (Moseley 1992, 5), that over deep prehistory along the Peruvian littoral, “the rich near-shore fishery had provided caloric support for 1) preceramic sedentary residence; 2) population growth, 3) large communities, and 4) the rise of complex societies which constructed very large architectural monuments before the advent of intensive irrigation agriculture.” It remains deeply significant, not least because it challenges—in one of humanity’s few pristine hearths of civilization—the “axiom that agriculture is necessary for the rise of complex societies” (Moseley and Feldman 1988, 125).

Although the MFAC hypothesis sometimes seems itself to have assumed axiomatic status for the Andean Region, it has in fact been subject to sustained criticism, leading even to pronouncements of its demise (*e.g.*, Haas and Creamer 2006). Such challenges are threefold:

1. Theoretical arguments that, relative to terrestrial food production, gathered marine productivity was inadequate, or inconsistent due to the periodic perturbations of the El Niño Southern Oscillation (ENSO), or insufficiently accessible to ancient exploitation, to support large sedentary populations (for reviews and refutations, see Quilter and Stocker 1983; Moseley and Feldman 1988; Quilter 1992).
2. More recent evidence that sophisticated maritime adaptations here go far further back in time than originally conceived in the MFAC hypothesis (*e.g.*, Sandweiss 2003; Arriaza *et al*. 2008; Dillehay *et al*. 2012; Lavallée and Julien 2012; Reitz *et al*. 2016a), thereby begging the question of why social complexity, if based upon marine resources, did not develop earlier (Sandweiss 2009, 39)
3. A still unfolding archaeological record for the monumental developments of the Late Preceramic Period (“Preceramic VI” or “Late Archaic Period”) in the 3rd millennium bc on Peru’s central (*e.g.*, Shady and Leyva 2003) and northern coasts (*e.g.*, Dillehay *et al*. 2012; Kaulicke 2012; Alva 2014), which suggests that these also entailed a “surge in the richness of food crop varieties” (Pearsall 2008, 113, see also Dillehay *et al*. 2004; Haas and Creamer 2006; Sandweiss 2009)

Yet, determining the validity of the MFAC hypothesis requires, as Aldenderfer (2006, 746) notes, an understanding of those changes in settlement dynamics and subsistence that *foreshadowed* the precocious developments of the Late Preceramic. That prelude, as the preceding long Middle Preceramic Period drew to a close, has been studied at only a few sites reported in any detail, notably in the Chillón (Lanning 1967; Patterson and Moseley 1968; Moseley 1975), Chilca (Engel 1987; Quilter 1989; Benfer 1990), and Zaña valleys (Dillehay 2011).

Our purpose in this article is to re-examine the MFAC hypothesis with findings from the south coast of Peru, a region hitherto lacking from the debate and whose Preceramic archaeological record has not been much investigated since the pioneering work of Engel in the 1950s. In particular, we present evidence from new excavations of two Middle Preceramic sites, La Yerba II and La Yerba III, at the mouth of the Río Ica, with occupations spanning the millennium from around 7000 to 6000 Cal bp. While subsistence at both sites was gained largely from marine resources, over time they evince emerging social complexity, defined herein by technological innovation, increasing sedentism, population density, ritual, social differentiation, and division of labor.

However, as we will see, this trajectory towards intensification and complexity never culminated on the south coast, as it did further north, in the eventual emergence of Late Preceramic monumental civilization, epitomized perhaps by the site of Caral in the Supe Valley. If the MFAC hypothesis holds, it should surely reflect and explain this stark contrast between different parts of the Peruvian coast, not least because, following the advent of agriculture, these same south coast valleys became the locus of some of the richest flowerings of Prehispanic societies during the Early and Late Intermediate Periods (*e.g.*, Willey 1971; Soßna 2015). Indeed, here the hypothesis appears to fail a test Moseley (1992) himself sets, for within the highly productive belt of nearshore Peruvian coastline, productivity maxima arise from upwellings at around 8°, 11°, and 15° South (Walsh 1981), with order of magnitude greater fishing yields of up to 1000 t/km^2^/year today. Moseley (1992, 22) would have it that these “productivity maxima … occur in the coastal region where the largest preceramic monuments were built.” Yet there are no monumental Preceramic constructions south of 12° S, and moreover, at La Yerba lying almost at 15° S, no Late Preceramic occupation at all.

The MFAC hypothesis has already evolved in response to its critics, not least through a renewed “calling to attention to the often ignored agricultural component of the original formulation” (Sandweiss 2009, 39; see also Moseley and Feldman 1988; Moseley 1992, 2005), in particular, of the fishing techno-cultivars of bottle gourd and cotton. As Sandweiss (2009, 50) observes, “the establishment of Caral and other inland Late Preceramic centers is best explained as an attempt to increase the production of cotton and gourds, to support intensification of … net-fishing.” Indeed, ever since Engel (1957a), some see cotton as so defining the Late Preceramic economy that they term this the “Cotton Preceramic Period” (Bird *et al*. 1985; Quilter 1991a, b; Pearsall 2008).

In this article, we explain the apparent failure of the MFAC hypothesis on the south coast of Peru by building on those earlier arguments to elaborate more explicit links than hitherto between the detailed technological aspects of marine exploitation using plant fibers to make fishing nets, including cotton, and the emergence of social complexity in the Andes. The story of civilization in Peru is, we will argue, a story of *nets*, shaped by Métraux’s (1959) powerful exegesis for how a *new* raw material can provoke a revolution in an *existing* technology, and thereby in society.

## The Preceramic Archaeological Record of the South Coast

The term “south coast” is defined herein as the 250 km of Pacific coastline encompassed by the Pisco, Ica, Río Grande de Nazca, and Acarí river valleys: a region geographically distinct from the rest of Peru’s long littoral both to the north and the far south (Beresford-Jones 2011), and which, for much of prehistory, enjoyed a distinctive cultural trajectory (*e.g.*, Willey 1971, 78). Here, following in the footsteps of Frédéric Engel (1957a, b, 1963b, 1981, 1991), Cambridge University’s One River Project has investigated an entire landscape of Preceramic archaeology along the littoral between 14° 19′ and 15° 15′ S. New excavations and surveys were carried out including sampling for flotation to extract organic remains and heavy fraction components, geochemistry, monoliths for micromorphology, radiocarbon dating, *etc.* (see Arce *et al*. 2013; Beresford-Jones *et al*. 2015a; Chauca and Beresford-Jones 2016).

**Fig. 1:**
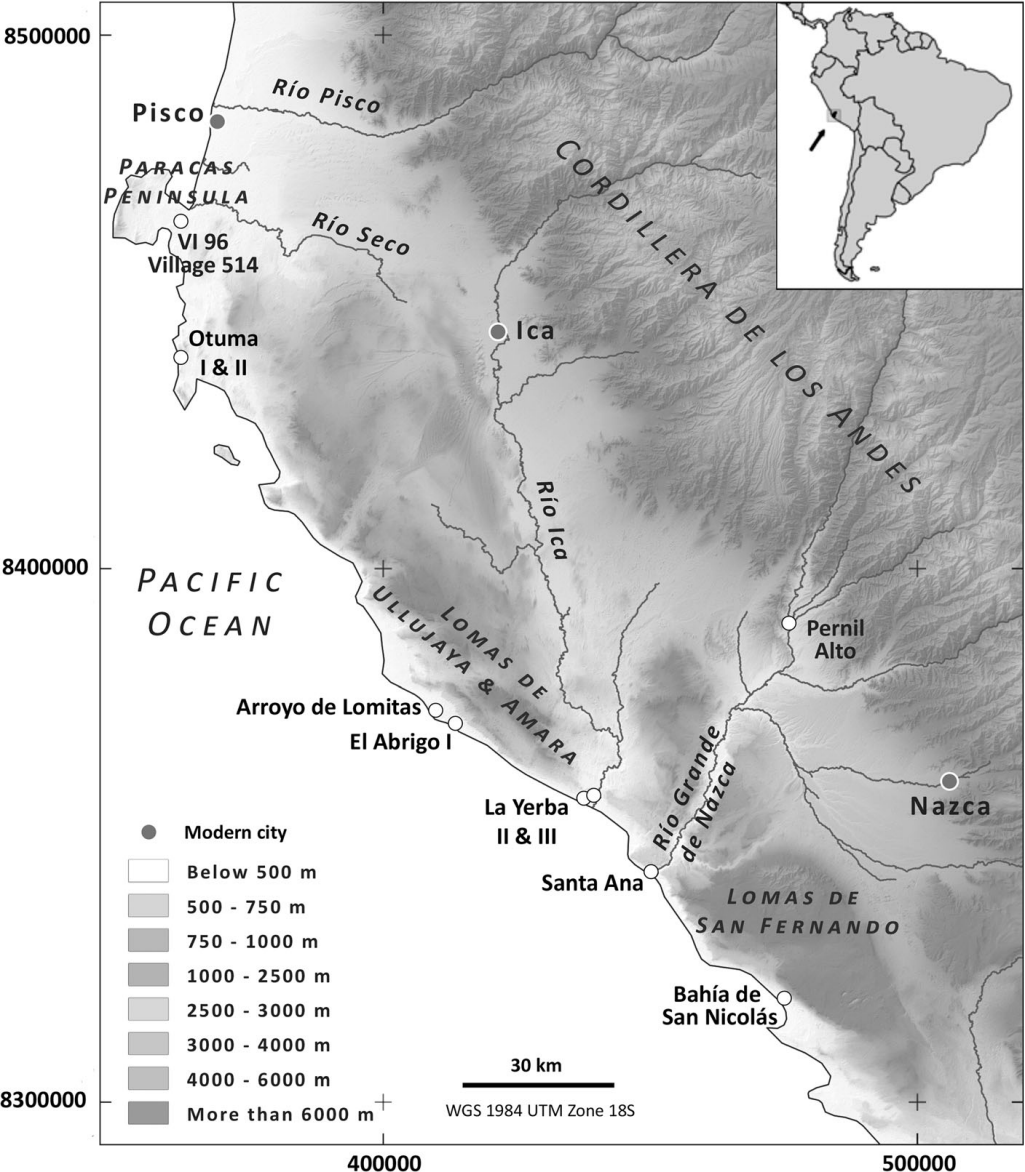
Preceramic sites on the south coast of Peru discussed herein

This archaeological evidence suggests that the south coast has been occupied at least throughout the Holocene. Thanks to a particularly narrow continental shelf, here some 60 m of sea level rise throughout the Holocene to 5800 Cal bp caused relatively little horizontal displacement of the shoreline (*e.g.*, Sandweiss 2009). Together with a complex history of ongoing tectonic uplift (Hsu 1992; Saillard *et al*. 2011), this means that older sites elsewhere inundated by the sea enjoy exceptional archaeological visibility. Sites such as the Abrigo I rock shelter (10,200–9539 Cal bp) and the “Visitantes” (or “VI 96,” 10,229–8074 Cal bp) stratigraphy underlying Village 514, Paracas (see Fig. 1, Engel 1991; Beresford-Jones *et al*. 2015b), suggest occupation here since the Early Preceramic.

**Fig. 2:**
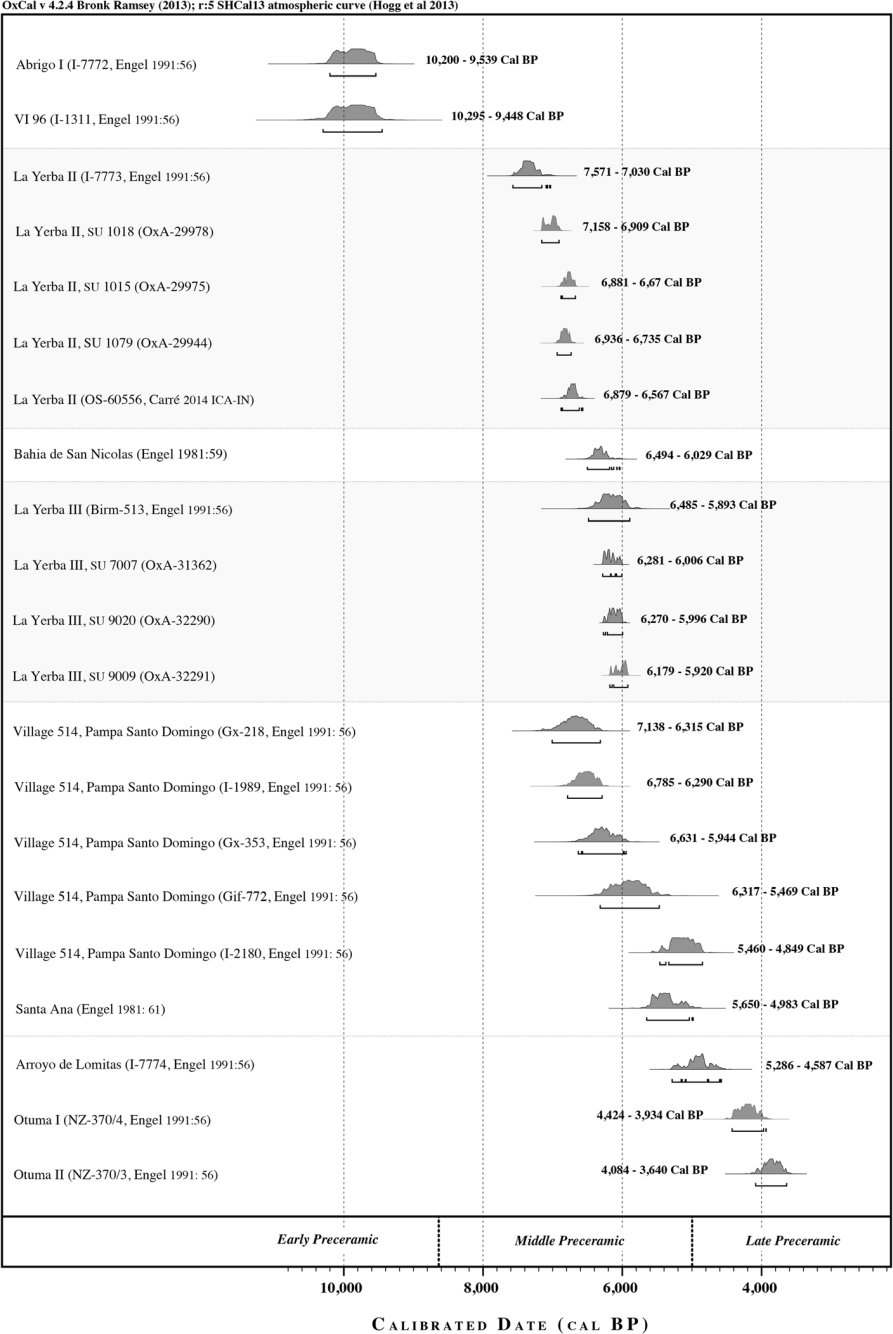
Radiocarbon dates for Preceramic sites discussed herein, all calibrated using the ShCal13 curve (Hogg *et al*. 2013) in OxCal version 4.2 (Bronk Ramsey 2009)

During the long subsequent Middle Preceramic Period, however, many more sites became established widely dispersed all along the littoral to exploit different marine and terrestrial ecological niches (see Fig. 2). This occurred once eustatic sea levels had stabilized and subsequent shoreline progradation had formed the beach habitats necessary for abundant, easily collected *Mesodesma* clams, and during a long epoch of, on average, colder seas, conveying increased intensity of coastal upwelling and more persistent ocean fogs (Beresford-Jones *et al*. 2015b). Here, as indeed elsewhere along the Pacific littoral of Peru and Chile (Benfer 1990; Lavallée and Julien 2012; Marquet *et al*. 2012), such favorable conditions for marine and ephemeral fog oasis environments seem to have enabled gradual increases in populations. Unsurprisingly, the largest and most visible of these coastal Middle Preceramic sites are found at the river estuaries, typified by two sites on the Río Ica estuary, La Yerba II and II, investigated here in some detail.

The earlier of these, La Yerba II^1^ (7571–6674 Cal bp), is a large shell midden some 175 m long and covering 9166 m^2^ to a depth of over 4.5 m on the east bank Río Ica (Arce *et al*. 2013). Largely composed of ancient surf clam shells (*Mesodesma donacium*) and dune deposits, the midden also contains lenses of occupation deposits comprising indurated ashy hearth rake-outs, made-ground surfaces, and the remains of areesh wind shelters, akin to those of historical Yaghan hunters-gatherers on Tierra del Fuego (*e.g.*, Lothrop 1928). Micromorphology reveals these to be vestiges of episodic occupations interrupted by inputs of windblown fine sands marking abandonment phases. When it was occupied, the La Yerba II shell midden was, like those of the Yaghan, situated considerably closer to the ancient surf line. Its position today, with basal deposits atop a relict marine terrace 17.9 m above mean sea level and overlooking a wave-cut platform almost a kilometer behind the actual shoreline, is, we suggest, the result of beach progradation following eustatic sea level stabilization around 5800 bp and ongoing tectonic uplift of perhaps 0.9 m per ka at the mouth of the Río Ica (Hsu 1992; Saillard *et al*. 2011).

The La Yerba II midden deposits are dominated by marine resources—mollusks, crustaceans, fish, kelp, and marine mammals—supplemented too with resources hunted and gathered from estuarine and *lomas* fog oasis environments, including Cyperaceae rhizomes, land snails, and hunted deer and guanaco (Arce *et al*. 2013; Beresford-Jones *et al*. 2015b). Manifestly La Yerba II was located at the convergence of different ecotones (*cf.* Arnold 1995) which, aside from the basic requirement of fresh water, provided efficient exploitation of the diverse resources of sandy beach, rocky headland, *lomas*, riparian woodland, and estuarine habitats. The La Yerba II archaeological assemblage suggests the vestiges of repeatedly reoccupied logistical base camps (*sensu* Binford 1980) of complex hunters-gatherers exploiting high-return foods according to seasonal rounds (*cf.* Ballester and Gallardo 2011).

**Fig. 3:**
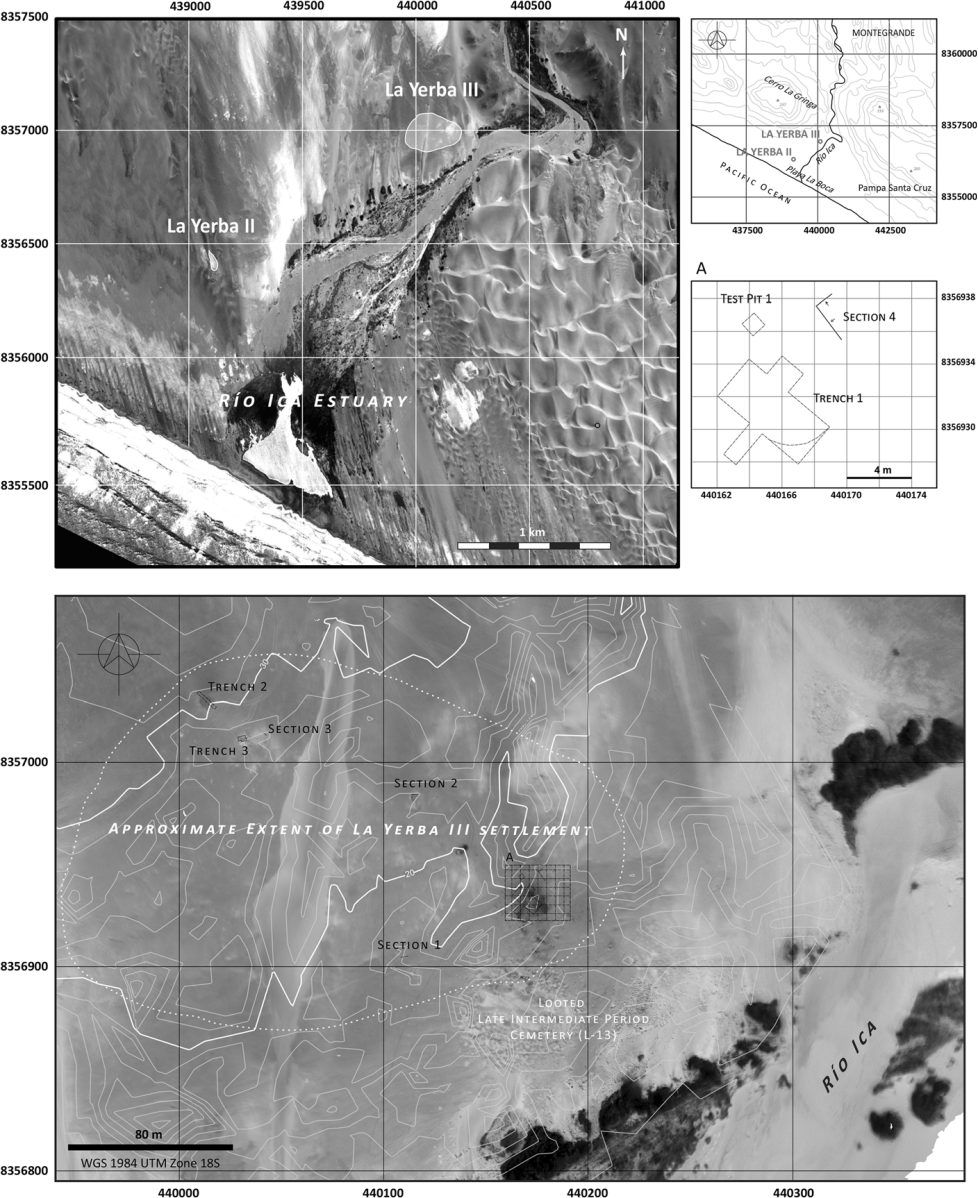
Río Ica estuary showing La Yerba II and III Middle Preceramic sites with details of excavations at La Yerba III (Chauca and Beresford-Jones 2016)

**Fig. 4:**
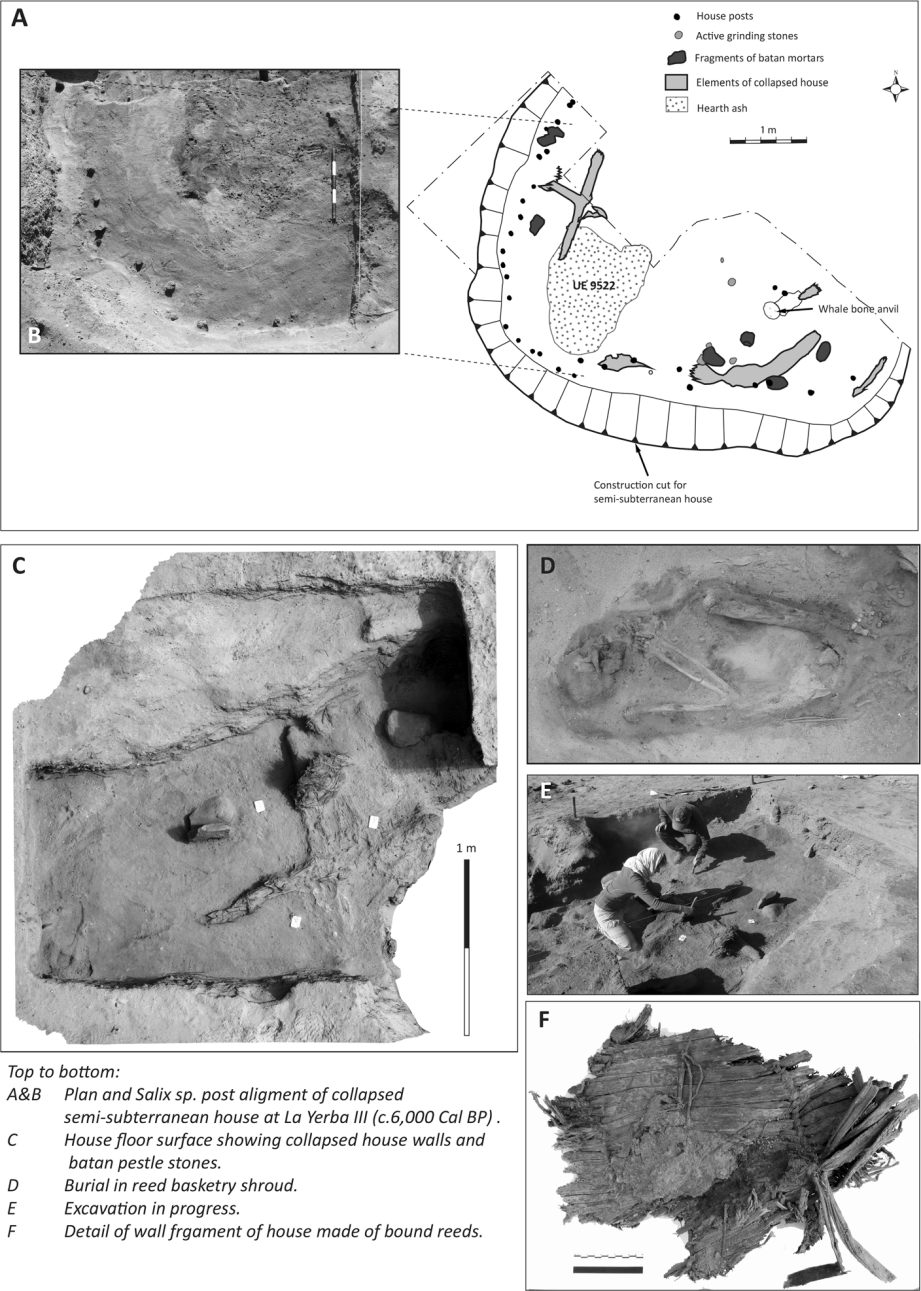
Selected details of excavations at La Yerba III (6485–5893 Cal bp), Río Ica estuary, south coast Peru

About a kilometer further inland along the Río Ica estuary is the slightly later site of La Yerba III (6485–5893 Cal bp), which presents striking contrasts to the La Yerba II shell midden, for this was a permanently, or nearly permanently, settled village over some 4 ha and composed of many superimposed semi-subterranean dwellings set amidst dense midden deposits with many structured human interments (see Figs. 3, and 4). Its population was an order of magnitude greater than its earlier neighbor La Yerba II, and it had much more non-portable site architecture, including well-made houses some 5 m in diameter, numerous large grinding stones, and storage pits. Rare in La Yerba II deposits, obsidian debitage was abundant at La Yerba III. These suggest spheres of interaction extending to the highlands since the compositions of all La Yerba obsidians are consistent with the well-known Quispisisa source at Huanca Sancos, Ayacucho, some 160 km as the condor flies from La Yerba (Glascock 2017). Just as they did at La Yerba II, marine foods underpinned the diet at La Yerba III, together with hunted and gathered wild faunal and floral resources from the local *lomas* fog oasis and river valley ecologies, but at La Yerba III there were also a number of important edible cultivars, including domesticated lima beans (“pallar,” *Phaseolus lunatus*), jack beans (“pallar del gentil” *Canavalia ensiformis*), guava (*Psidium guajava*), and animals in the form of guinea pigs (*Cavia porcellus*) and dogs (*Canis familiaris*).^2^

This trajectory towards increasing intensification and more permanent village settlement at La Yerba is reflected along the wider south coast from around 6500 Cal bp (during “Preceramic V”) at other shoreline villages such as Paloma and Chilca I, Chilca Valley (Donnan 1964; Engel 1970, 1987; Quilter 1989, 1991b; Benfer 2008), and more perfunctorily reported sites of Village 514 (or “Algodón y Jíquima”), Paracas, and Santa Ana on the Río Grande de Nazca estuary (see Figs. 1 and 2, Engel 1960, 1981, 1991, Nicho *et al*. 2016).

All along the south coast, therefore, the end of the Middle Preceramic Period saw the establishment of sedentary or near sedentary villages such as La Yerba III whose subsistence bases were still dominated by maritime resource exploitation, but which reflected also those changes, widely recognized to precede the emergence of agriculture in many parts of the world, for which Flannery (1969) coined the term “Broad Spectrum Revolution.” At La Yerba, this included a widening use of resources to include floodplain farming and perhaps animal husbandry, more extensive trade or exchange networks, and structured mortuary deposition connoting territoriality.

Moreover, these south coast trajectories were paralleled by a much wider shift to more permanent village settlement, epitomized by those sites on the central coast that Moseley first used to formulate the MFAC hypothesis: at the Yacht Club and Tank sites at Ancón, Chillón Valley (Lanning 1967; Patterson and Moseley 1968; Moseley 1975).

Further inland, the archaeological record for the Preceramic on the south coast is scarce, as indeed it is elsewhere, perhaps obscured by subsequent agricultural and geomorphological changes (Engel 1981; Isla 1990; Cook 1994; Vogt 2011). Some 60 km inland near Palpa, however, a Middle Preceramic village called Pernil Alto is reported in some detail (Gorbahn 2013, see Fig. 1). Settled ca. 5800 Cal bp and perhaps occupied year round, its occupants carried out horticulture of lima beans, sweet potato, gourds, common beans, achira, jack beans, and guava. Here, some 60 km from the littoral, marine resources were apparently not significant to diet, though the presence of some mollusks indicates contact with the coast, lying no more than 2 days walk distant. The south coast hinterland was, therefore, undergoing horticultural settlement by the end of the Middle Preceramic, and indeed widening socio-economic relationships between coastal, piedmont, and highland Andean communities are suggested by the presence of marine shell inland and the large quantities of highland obsidian found at coastal sites like La Yerba III and Santa Ana (Beresford-Jones *et al*. 2015b, Glascock 2017).

Yet, the single most striking feature of the Preceramic archaeological record of the south coast is that after 5000 Cal bp, the date more widely construed to mark the dawn of the Late Preceramic elsewhere in Peru,^3^ there are very *few*, and only ephemeral, occupations along its littoral. Even its river estuaries seem to be abandoned (see Fig, 2, Engel 1981, 1991; Fung 1988; Carmichael 1991; Beresford-Jones *et al*. 2015b). This “archaeological silence” may mirror significant population declines evidenced by using summed radiocarbon probability densities as a proxy, from archaeological sites from coastal southern Peru and northern Chile, extending south to 27° S (Marquet *et al*. 2012). On the south coast of Peru, it is interrupted by only a single apparent exception: idiosyncratic middens of scallop shells (ca. 4150 Cal bp) at Otuma, 25 km from Paracas, dating to between ca. 4424 and 3451 Cal bp (calibrating six dates given in Engel’s 1991: 56, and see Craig and Psuty 1971: 130).

## Fishing Technology at La Yerba II and III

Excavations at La Yerba II and II yield copious evidence of marine resources through a combination of fine gauge dry sieving and flotation of targeted deposits (see Arce *et al*. 2013; Chauca and Beresford-Jones 2016). Among those, comparative analysis of fish bone remains in the archaeological contexts of La Yerba II and La Yerba III can be used to make certain technological inferences relating to fish procurement (*e.g.*, Reitiz *et al*. 2016b). Moreover, extraordinary preservation conditions for organic artifacts in the region’s arid climate open a window *directly* onto the trends in plant fiber fishing technologies that were entailed in the transition to sedentary living evident in the wider archaeological records of the two sites.

### Inferences for Fishing Technologies from Fish Remains

**Fig. 5:**
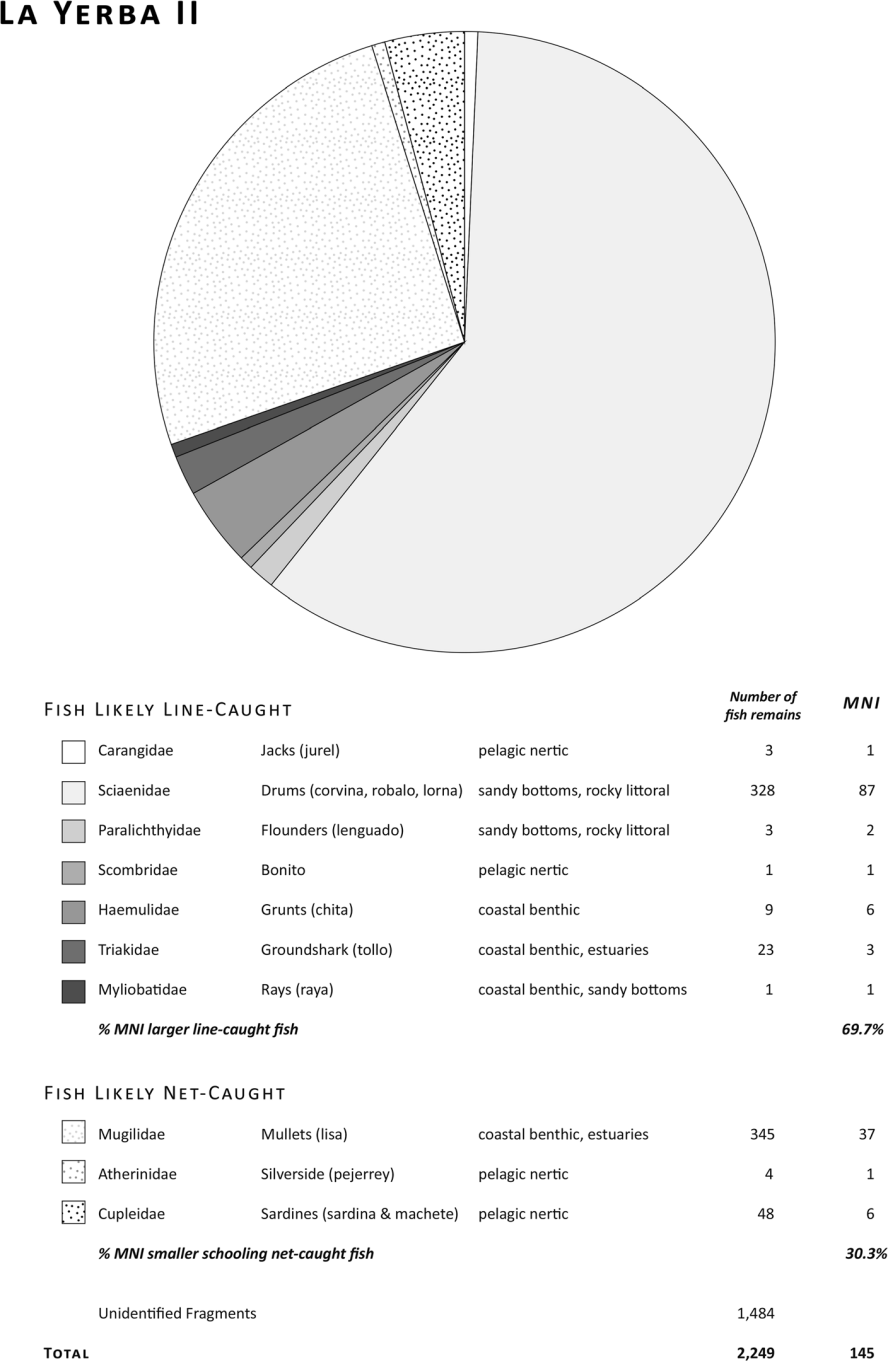
Fish remains at La Yerba II (7571–6674 Cal bp)

Fish remains from both sites comprise benthic and pelagic coastal or neritic species. At La Yerba II (see Fig. 5), 60% of identified fish remains by Minimum Number of Individuals (MNI) are of various species of drum (Fam. Sciaenidae): corvina (sea bass, *Cilus gilberti*, 44% by MNI), lorna (*Sciaena deliciosa* and *S. callaensis*, 9% by MNI), robalo (*Robaloscion wiener*, 2% by MNI), and coco (*Paralonchurus peruanus*, 5% by MNI). Twenty-five percent by MNI are of mullet (lisa, *Mugil cephalus*), and there are minor percentages of sardine (*Sardinops sagax*), and chita (*Anisotremus scapularis*), and occasional pejerrey (*Odontesthes regia*), jurel (*Trachurus murphyi*), machete (*Ethmidium maculatum*), lenguado (*Paralichthys adpersus*), bonito (*Sarda chiliensis*), rays (*Myliobatis* sp.), and sharks (tollo ground shark, *Triakis* sp. and blue shark). The La Yerba II contexts also contained a few remains of bottle-nosed dolphin (*Tursiops truncates*) and bones of indeterminate whale species. Notably, there were few anchovy (*Engraulis ringens*) remains at La Yerba II. Their bones are very small and thus likely under-represented in most recoveries; yet, even flotation of 46 l of La Yerba II contexts yielded an MNI of only four anchovies.

**Fig. 6:**
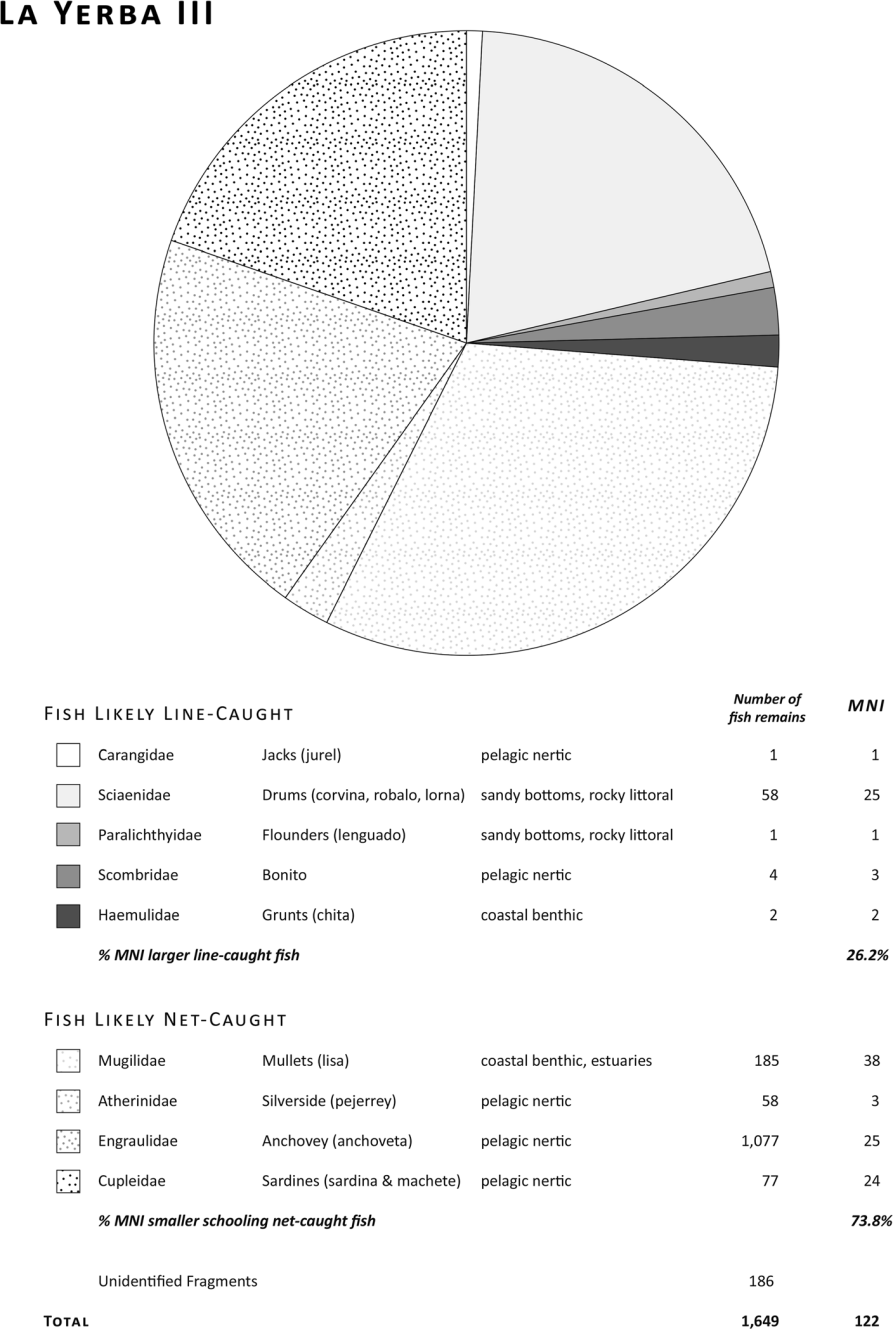
Fish remains at La Yerba III (6485–5893 Cal bp)

At La Yerba III (see Fig. 6), meanwhile, the most abundant species are lisa (31% by MNI), anchovies (21% by MNI), and sardine (19% by MNI). There are also significant percentages of corvina, lorna, robalo, bonito, and pejerrey, together with occasional jurel, machete, lenguado, and chita.

Because of their small jaws and feeding behavior, some fish such as lisa are notoriously difficult to target using hooks, but relatively easy to capture with nets. Others such as sardine, anchovy, and pejerrey must be captured with some sort of netting because of their small size and feeding habits (sardine, for instance, are filter feeders). But since such small fish may also form the gut contents of larger carnivores, their mere presence in archaeological contexts does not, of itself, verify the use of nets.

**Fig. 7:**
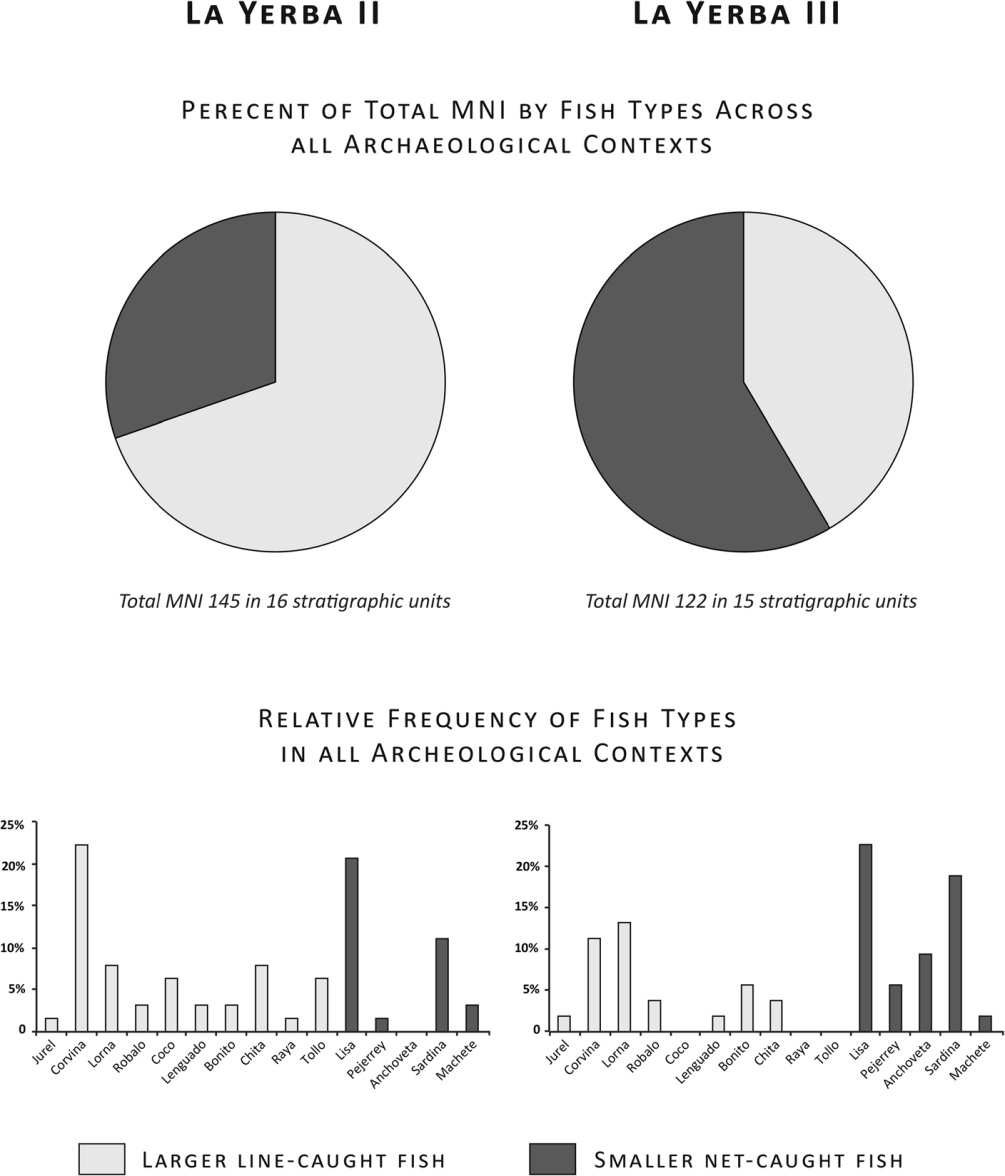
Comparison of fish remains at La Yerba II and III

Significantly increased relative frequencies overall of anchovy, sardine, pejerrey, and mullet at La Yerba III (see Fig. 7), however, do suggest more fishing using nets of small mesh sizes (ca. 10 mm) than at La Yerba II (*cf.* Moseley and Feldman 1988). The relative frequency of anchovy, doubtless under-represented in recovery, suggests that this fish had become a target species by this time, rather than being introduced incidentally to these contexts. Although larger fish such as corvina, chita, or lenguado may also be landed with small gauge nets, these predatory species are more easily taken with lines using baited lines, or with harpoons. Today, corvina and other large fish are taken with both gill nets and hook and line from the beach. Larger fish in the contexts of both sites, such as frequent corvina to 9 kg in weight, and fewer sharks and perhaps dolphins, were likely captured offshore with baited lines. Indeed, certain species such as anchovy, bonito, and blue shark are generally found some distance offshore (ca. 0.5 km) in deeper water, though their presence in the La Yerba assemblages is too small to argue convincingly for fishing using some form of watercraft.

Though many of the smaller fish like pejerrey, machete, and juvenile sardine can be found nearshore year round, it is notable that the shoreline availability of the main target species at both La Yerba sites fluctuate seasonally. Today, the shore fishing season at the mouth of the Río Ica is during austral spring and summer (October through February), the season referred to here as the “bonanza.” In winter, winds are stronger, the sea is rougher, and key target species are absent, or much less concentrated, rendering shore-based fishing difficult or unproductive.^4^ In spring, when the intermittent rivers flow, lisa come into spawn in the estuaries and later can become cut off from the sea by falling river levels and concentrated in large numbers, whereupon they are easily caught using cast nets or by driving them into small nets. Single landings in this way of up to 200 kg of lisa are reported in the Río Ica estuary today. By late summer in February, freshwater crayfish in the river’s outwash becomes an important source of food, bringing hunting corvina close inshore to the estuary. But exceptionally strong river flows during summer can bring so much debris that net fishing at the estuary becomes impossible. During the warmer summer weather, both anchovy and sardine shoal much closer to the shore, so that even these species are seasonal when fishing takes place from the shore (Gutiérrez *et al*. 2007).

Rare, powerful El Niño events on the south coast such as that of 1997/1998 bring warmer waters that can drive huge numbers of anchovy close inshore together with corvina and other predators. Gorged on prey borne on a flooding river during such events, fishermen report corvina to become “stupid” and far easier to catch in greater numbers. Meanwhile, populations of cold water mollusks such as surf clams can collapse. The effects of El Niño on fishing productivity are therefore far from straightforward (cf. Moseley 1992: 15).

### Direct Evidence of Fishing Technologies

**Fig. 8:**
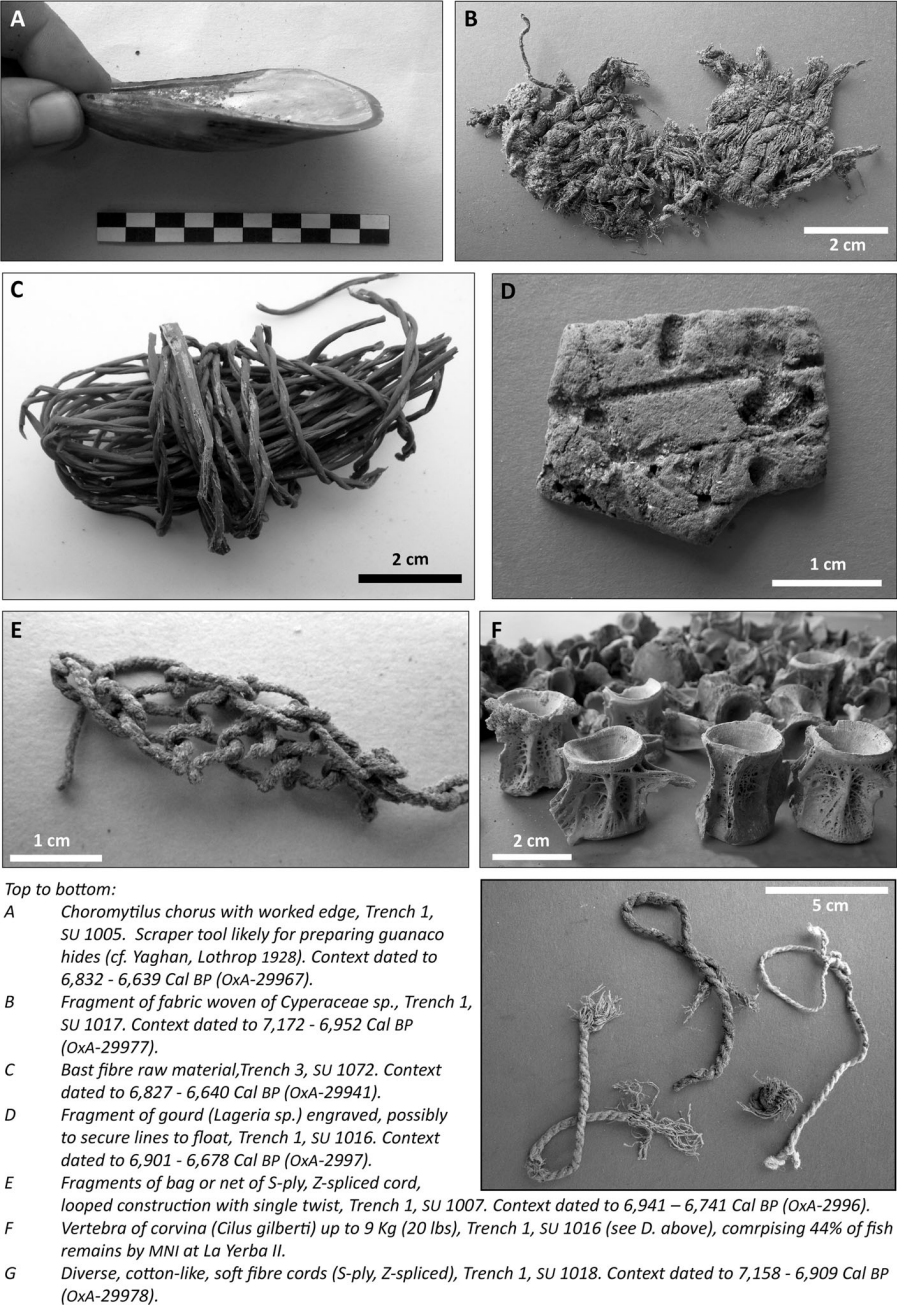
Selected finds from La Yerba II

**Fig. 9:**
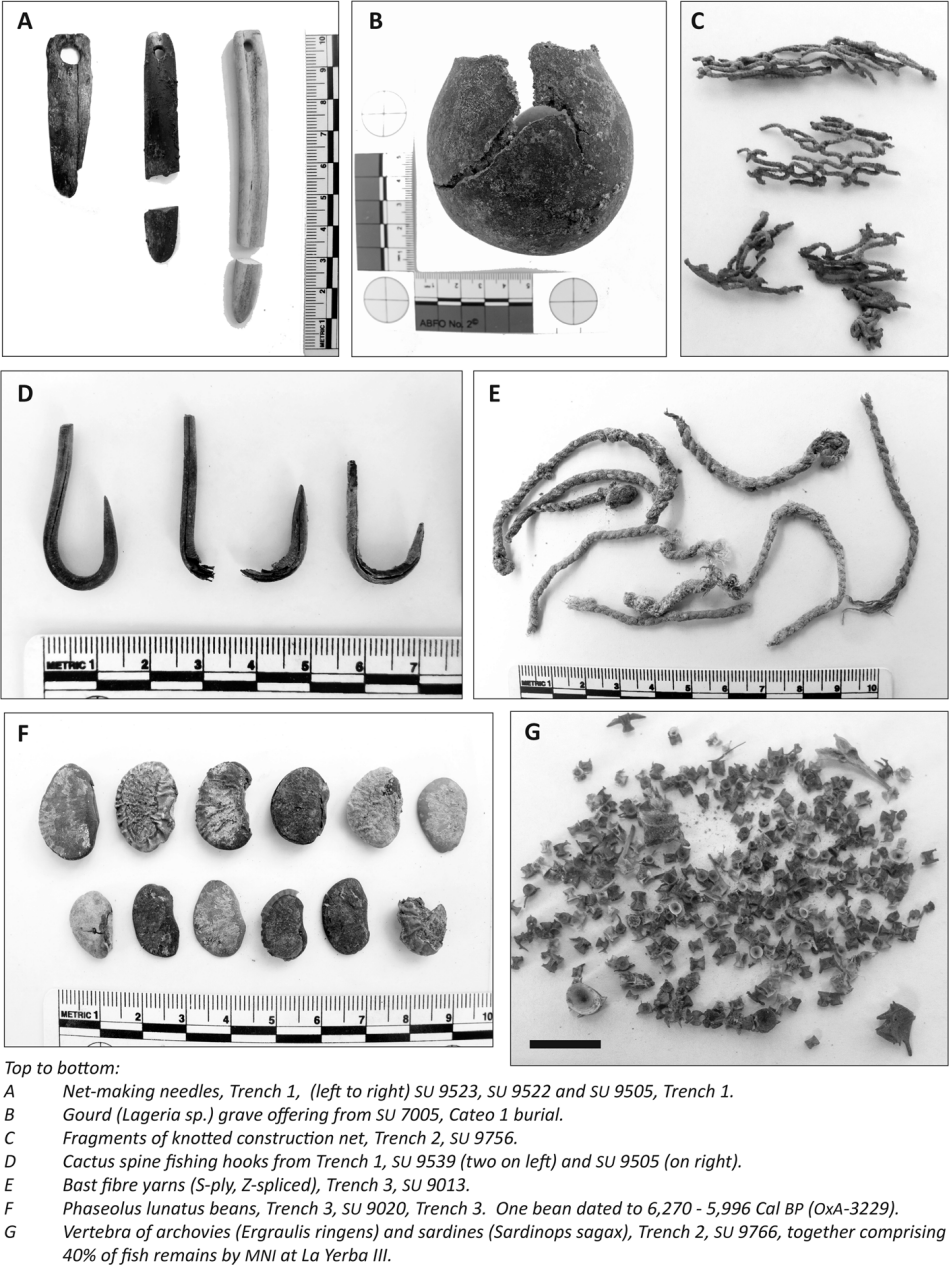
Selected finds from La Yerba III

Fishing can be carried out in three basic ways: using harpoons, lines, or nets (Cushing 1988). The contexts of La Yerba II and III contain many vestiges of such equipment (see Figs. 8 and 9), including: gorges, for which there is direct evidence of use for line fishing back 10,000 years in the New World (Rick *et al*. 2001); harpoons at La Yerba II; and fish hooks formed from cactus spines at La Yerba III. Fragments of bottle gourd (*Lagenaria* sp.) are frequent at both sites and may be evidence of floats used for the beach seine nets known by the Late Preceramic (see Bird *et al*. 1985), though they may also be the remains of diverse containers. Most obviously, however, evidence for fishing here was in the form of copious cordage, lines, and, occasionally, fragments of net.

Different plant fibers, sometimes in combination, were used to make these. Thicker cordage seems to have been produced from *Cyperaceae* sp. (“junco”) stems, but many finer, lighter yarns are common at either site and are more suited to production of fishing lines or nets for which, among other technical characteristics, reduced visibility is important. These finer yarns, the vast majority z-“spun” (or more correctly “spliced,” see below) and s-plied, vary in color between white and shades of brown and to the naked eye can easily be mistaken for cotton (Bird *et al*. 1985, see Figs. 8 and 9). Lanning, for instance, in Vescelius (1963, 44), claims “remains of gourds, cotton and cotton netting” in excavations made with Engel at La Yerba II^1^. Yet, our scanning electron and light microscopy analyses of yarns from La Yerba II and III affirm that they are not made of cotton, but rather of plant bast fibers. Moreover, these yarns were not spun but rather spliced: a critical technological distinction, the implications of which will be discussed later. Nor do we identify any parts of cotton plants such as seeds in flotation of midden contexts from these sites.

**Fig. 10:**
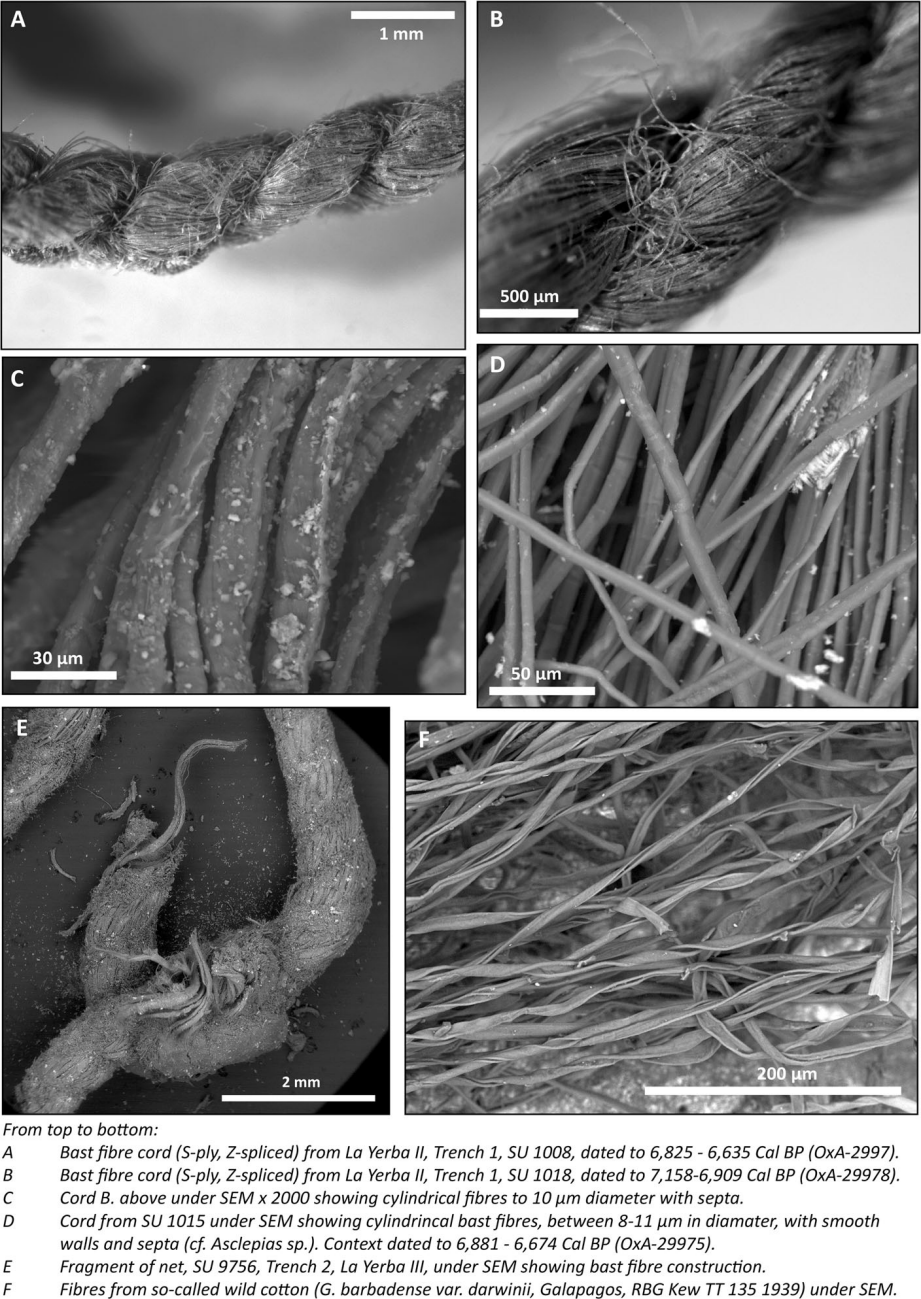
Selected details of yarns and cordage from La Yerba II and III

In the Old World, bast fibers from under the outer cortex of the stems of plants such as linen, hemp, nettle, and jute have long been an important source of yarns and cords of very diverse characteristics. Following the retting processes by which such fibers are extracted, these can be difficult to distinguish taxonomically. Our analyses (see Fig. 10) suggest that the bast fiber yarns at La Yerba are likely of to be of *Asclepias* spp*.* (“milkweed”), or a closely related plant of the dogbane (Apocynaceae) family (Endress *et al*. 2014). This family includes a number of climbing vines of the Tribe Asclepiadeae native to Peru, such as *Asclepias curassavica* and *Funastrum clausum*, common today in the Ica Valley. *Asclepias* spp*.* seed pods have been reported at a number of Preceramic sites on the central (Patterson and Moseley 1968; Cohen 1978) and north coasts of Peru, including at Huaca Prieta (Bird *et al*. 1985; Splitstoser *et al*. 2016). As hinted at by vernacular names such as “white twine vine,” “silkweed,” and, in Peru, “algodóncillo” (little cotton) and “flor de seda” (silk flower), plants of the Apocynaceae family have long been an important resource for fiber production throughout the New World (Whiting 1943).

In Peru, *Asclepias* sp. vines themselves are sometimes used as lashings (Bird *et al*. 1985), but the plant offers two potential sources of fibers. The silky seed floss of *Asclepias* sp. superficially resembles the fibers in a cotton boll, although these fibers are relatively short and brittle, only producing adequate yarns in combination with other fibers (Whiting 1943; Reddy and Yang 2009). As we will discuss shortly, however, *Asclepias* sp. stems also produce excellent, fine bast fibers.

Fragments of netting material with small mesh sizes up to 12 mm were recovered at both La Yerba II and III. At the former, only a single fragment of looped net with a single twist was found (see Fig. 8E). By contrast, at La Yerba III multiple fragments of mesh from nets are knotted: a significant development in net technology, preventing mesh from slipping or nets from distorting and enabling larger nets (see Fig. 9C).

The looped, twisted net fragment from La Yerba II may or may not have been used for fishing. Certainly, it would have been less prone to distortion than a simple looped net, which needs to be stretched on a frame. Meanwhile, the knotting technique evident at La Yerba III clearly represents an increased investment in the time of net production (Gabriel *et al*. 2005). That investment and the improvement in net making at La Yerba III is perhaps best evident in the bone net-making needles recovered here (see Fig. 9A), which would have speeded up and standardized net production (Gabriel *et al*. 2005). By the Late Preceramic, knotting would have become essential to net making because smoother cotton yarns, more prone to slip than bast fibers, would be less effective for looped and twisted nets.

## Discussion

Civilization first arose in the Late Preceramic Period during the 3rd millennium bc in several valleys of the Peruvian coast. While still typified by the extraordinary agglomeration of monumental sites at Caral in the Supe Valley on the central coast (*e.g.*, Shady and Leyva 2003), there are now increasing suggestions of even earlier planned monumentality at sites further north (Dillehay *et al*. 2012; Kaulicke 2012; Alva 2014). It is no surprise, then, that the *Maritime Foundations of Andean Civilization* hypothesis was first formulated from a reading of the archaeological record of Peru’s central coast (Moseley 1975, 3), the most southerly expression of which are the monumental sites of the El Paraíso tradition, Chillón Valley, near Lima (Quilter *et al*. 1991; Quilter 1992; Cornejo 2013). South of around 12° S, the archaeological record of the Late Preceramic seems to include only modest sites lacking any monumental architecture, such as that at Asia Unit 1 (Engel 1963a). On the south coast of Peru, there was manifestly *none* of the Late Preceramic florescence of greater population densities and monumental civilization that occurred further north (*e.g.*, Fung 1988: Fig. 3.1), thereby begging the question of *why* the MFAC hypothesis appears to have miscarried here, and what that may imply for the hypothesis.

### Intensification on the South Coast

For along all the south coast littoral as defined herein, the only significant archaeological site dating to the Late Preceramic is the idiosyncratic site of Otuma, 25 km south of Paracas, composed of enormous, almost monotypic middens of *Argopecten purpuratus*, set about a now desiccated lagoon (Engel 1957b, 1981; Craig and Psuty 1971). Presumably, these were ecologically restricted to those warmer lagoon waters because in the La Yerba assemblages we record only a single *A. purpuratus* shell, in the form of an artifact with a rounded and beveled lip. Fishing nets, reported in some quantities at Otuma, provide the earliest unequivocal evidence^5^ from the south coast for the use of cotton for fishing technology at around 4150 Cal bp. Yet aside from Otuma, and even at the river estuaries, this littoral is seemingly marked by a profound archaeological silence during the Late Preceramic (see Fig. 2): a quiet which, as already noted, seems to be reflected in population declines at this time extending far south to 27° S in northern Chile (Marquet *et al*. 2012).

There is no plausible reason to suppose that the stark contrast here with the precocious flowering of monumental civilization further north can be explained by any relative poverty in marine resources. ENSO perturbations can impact marine resources, not least the population of *Mesodesma* clams, a key dietary component at La Yerba, but such perturbations would far more greatly affect seas further north (El Niño being a north to south phenomenon), and the latest reconstructions suggest a substantial *reduction* of ENSO variance between 5000 and 4000 bp (Carré *et al*. 2014). Today, the richest concentrations of anchovy along western South America extend south to 16° S, and upwellings off 15° S make the south coast waters among the most productive in Peru (Walsh 1981; Moseley and Feldman 1988; Gutiérrez *et al*. 2007).

It is rather a contrast between the abundance of the *terrestrial* resources that determine any society’s capacity to intensify exploitation of maritime resources that underpins these contrasting cultural trajectories between these parts of the Peruvian coast: a distinction long-recognized in their respective geomorphological configurations (Kroeber 1944, 25; Engel 1981, 11; Quilter 1992, 118; Moseley 1992, 23). For unlike the river valleys to the north, with their broad alluvial deltas and wide ocean frontages granting easy access simultaneously to rich marine and agricultural resources, the rivers of the south coast comprise scattered floodplains down long courses, diverted and separated from the sea by a coastal cordillera rising in places to almost 2000 m asl.

Moreover, terrestrial river hydrologies may have been particularly impacted by a substantial reduction in ENSO variance at this time (Carré *et al*. 2014; Beresford-Jones *et al*. 2015b), or by an onset of extreme aridity (*cf.* Marquet *et al*. 2012). The corollary of intensifying marine resource exploitation through increased specialization in fishing was, counter-intuitively, an increased demand for *terrestrial* resources, which on the south coast were highly circumscribed not least by differential river hydrologies and set relatively further from the sea. This explains the apparent contradiction of the MFAC hypothesis here, and calls in turn for some necessary refinements to precisely how the MFAC unfolded, as follows:

Firstly, the broadening spectrum of resources evident at La Yerba III and elsewhere and the subsequent abandonment of the littoral in the Late Preceramic each imply that, rather than being incompatible specializations as hitherto often assumed, fishing and farming were likely carried out within the *same* society and, indeed, that it is their *compatibility* that lies at the heart of the MFAC hypothesis. Reitz *et al*. (2008, 129) assert that “the complexity of making a living safely and reliably from the sea calls upon tools, skills, and knowledge that are incompatible with part-time fishing” (see also Moseley 1992: 22). We disagree. Ethnographies of modern fishing societies show just how seasonal and often part-time fishing can be (Quilter 1992; Tietze 2011), not least for shore fishermen at the mouth of the Río Ica today. Increasing sedentism and social complexity at La Yerba through the Middle Preceramic went hand in hand with an intensification of fishing using nets, evidenced by increasingly sophisticated forms of netting, the appearance of net-making needles (see Fig. 9), and increases in the relative abundance of small schooling fish, such as anchovy, in the archaeological assemblages (see Fig. 7). This intensification of shore-based fishing would occur during the summer bonanza, when both fishing and processing (*e.g.*, salting and drying) were most productive. Inactive periods before and after would be used by a sedentary population for the planting and harvesting of food and industrial crops such as beans and gourds, and for targeting less seasonal marine species. Extended families pursue just such seasonal occupations today across the lower courses of the Río Ica.

Secondly, the fact that the sites at the mouth of the south coast rivers were abandoned *before* the dawn of the Late Preceramic elsewhere suggests that this long process of intensification stalled here because their circumscribed estuarine environments, lying relatively distant from upstream riparian basins, were vulnerable to over-exploitation of gathered wild plant resources for fuel, housing, and, critically, the bast fibers that underpinned their maritime economies. This is hinted at the Río Ica estuary by a greatly expanding sedentary population over the millennium between La Yerba II and III, alongside edible starchy rhizomes of estuarine *Cyperaceae esculentus* (“chufa”) in such quantities that they virtually define the occupation contexts of the former, while at the latter they are almost entirely absent.

As greater strain was perpetuated on wild terrestrial resources over generations, permanent sedentary living at the Río Ica estuary may have become increasingly difficult. A long-term response would be to invest more in horticulture and relocate upstream into the wider riparian basins in which resources such as fuel and fiber plants were more resilient to exploitation. Following abandonment of the littoral, inland horticulturalists would have continued to exploit the sea directly at logistical field camps (*sensu* Binford 1980), such as Arroyo de Lomitas (ca. 5286–4587 Cal bp, see Figs. 1 and 2 and Beresford-Jones *et al*. 2015b), though, because of increasing distances involved, marine resources would have formed far smaller components of the diet, just as at the site of Pernil Alto (Gorbahn 2013; Soßna 2015; and see also Engel 1981).

Lastly, we have seen how over the millennium to ca. 6000 Cal bp the La Yerba archaeological record entails much increased populations, more sedentary village lifestyles, wider exchange networks, a broadening spectrum of resources including, floodplain farming of beans and other food plants, and an intensified and increasingly sophisticated exploitation of maritime resources. All these changes, however, took place in the context of a fabric technology based upon plant bast fibers. Cotton, in time the preeminent plant fiber cultivar of the Peruvian coast, was therefore, when it eventually arrived, planted into an already unfolding scenario of sedentism, intensifying marine resource subsistence and incipient agriculture. These were people who *already* cultivated gourds and beans, and whose sophisticated plant fiber technology already granted access to the dryable, storable protein surplus represented by the vast anchovy stocks in Peruvian seas. They were, therefore, *pre-adapted* to what we will define as a “Cotton Revolution.”

Thus, while cotton has long held a central place in the MFAC hypothesis (Moseley 2005; Haas and Creamer 2006, 754), we seek here to elaborate hitherto unarticulated reasons for precisely how a transition in raw materials from bast fibers to cotton could have provoked a socio-economic revolution that laid the Maritime Foundations of Andean Civilization. This entails, in the first instance, consideration of the relative physical properties and modes of production of these plant fibers for the making of fishing lines and nets.

### Bast Fibers *vs.* Cotton

Long-staple *Gossypium barbadense* cotton, the product of domestication on the “northern and central coasts of Peru” (Stephens and Moseley 1973; Chaudhary *et al*. 2008), is today one of the major commercial annual crops of the Ica Valley. So-called primitive cultivar (“dooryard”) and wild *G. barbadense* cottons are mentioned in the literature as occurring in the dry coastal areas of the Gulf of Guayaquil, Ecuador, though, as Westengen *et al*. (2005, 393) note, they “are not clearly defined in terms of fitness-related-traits for wild survival.” Indeed, feral, dooryard or hybridized varieties of *G. barbadense* grow as part of the natural vegetation throughout its western South American native habitat, so that here no less a botanist than Richard Spruce had “perhaps nowhere seen a cotton plant truly wild” (cited by Watt 1907, 215). The coastal valleys of Peru are therefore the presumed natural habitat of a *G. barbadense* “wild-to-domesticated continuum” (Westengen *et al*. 2005, 393).

Yarns spun from *G. barbadense* cotton are excellent for fishing nets and lines because of a combination of strength and fineness. Strong, slender cotton yarns are light, enabling larger, more portable nets; give up moisture easily, limiting the rotting to which all damp organic fibers are subject; and, just like flax used to make nets in the Old World, become stronger when wet with seawater. Fishing nets are commonly dyed to increase their invisibility to fish, but it is relatively difficult to dye cotton with natural dyes and mordants. Peruvian native cottons, however, famously occur in a range of natural colors, including brown, gray, and green, which we speculate may have been originally selected for, for precisely this reason.

Nonetheless, purely in terms of the *technical* characteristics of its yarns for fishing, *Asclepias* bast fibers enjoy most, if not all, of the strengths of cotton. For they too produce fine yarns with greater breaking and tensile strengths than cotton, though they are not as quite as flexible or lightweight (Whiting 1943; Reddy and Yang 2009; Thevs *et al*. 2013). In the Old World, fishing technologies were likewise long dominated by plant bast fibers such as flax and hemp. *Asclepias* bast fiber has a higher breaking elongation than flax or other bast fibers (Whiting 1943, 8; Reddy and Yang 2009, 2216). And, just like cotton, *Asclepias* bast fibers increase their strength underwater making them particularly suitable for nets and fishing cord. *Asclepias* bast fibers were therefore perfectly sufficient to sustain an intensification of marine resource exploitation through the Middle Preceramic. The great difference between them and cotton as a source of raw material for fishing technologies lies *not* in their respective physical properties but rather in all stages of their modes of *production*.

Firstly, preparation of bast fiber raw material calls for far more labor than cotton (Goulding 1917), entailing as it does various processes, including splitting, rolling to separate the fibers from cortex, and buffing to separate the individual fibers (Bolley and Marcy 1907; Goulding 1917; Whiting 1943). If bast fibers are to be spun, as opposed to spliced as discussed shortly, they must be freed from gummy pectin and attached stem cellular tissues—usually by retting in water for periods of weeks. By contrast, the only labor-intensive part of the preparation of raw cotton before spinning is the removal of the seeds.

Secondly, the microscopic features of the La Yerba yarns strongly suggest that they were not spun but rather spliced on the thigh: a method that predates spinning for making yarns from plant bast fibers everywhere that there are extant records, in Egypt until the Middle Kingdom and in Europe through the Late Neolithic into the Bronze Age (see Fig. 10, Tiedemann and Jakes 2006; Gleba and Harris forthcoming). Moreover, no obvious spindles or spindle whorls are evident in the La Yerba sites (or indeed, to our knowledge, elsewhere in the Peruvian archaeological record until the Late Preceramic). Splicing is suited to longer bast fibers, but only around half as fast as spindle spinning. Indeed, we suggest that spinning was almost certainly a later technical innovation *necessary* for the making of yarn from the relatively shorter fibers of cotton. Perhaps innovated from using camelid fibers, which likewise cannot be spliced, spinning, once achieved, makes for a far more efficient and continuous process for producing yarn since it entails no need to “splice” individual bast fibers together.

Finally, the most significant difference by far between plant bast and cotton fiber technologies in the context of the Peruvian Preceramic is that the former is supplied from wild plants *gathered*, and in the process removed, from the local vegetation, whereas the latter is produced from a *cultivated* crop: a crop moreover which, in its natural habit of perennial “tree cotton” shrubs, requires little effort, beyond annual pruning to enhance bushy growth and inhibit fungal damage, to produce enormous quantities of fiber for harvest throughout the year (Goulding 1917). While a wild plant resource such as *Asclepias* vines might perhaps be managed to some extent, they would never offer ancient spinners and net makers anything like the control over the *supply* of fiber raw material as could cotton (*cf.* Sandweiss 2009, 50).

For just these reasons, early twentieth century efforts to industrialize *Asclepias* sp*.* as a source of fiber foundered. Whereas the *quality* of the yarns and textiles it produced were comparable to linen, it could never compete with cotton for *quantity* of production, and thereby price (Whiting 1943). For reasons of production too, cotton almost completely replaced flax as the primary raw material net manufacture in European fishing industries by the end of the nineteenth century (Bremner 1869; Ono 2010). Indeed, in Peru, cotton was only supplanted by nylon for nets in the 1950s, well into the modern era of industrial fishing.

Just as production factors of ease of raw material supply came into play millennia ago as the Middle Preceramic drew to a close and increased, more sedentary populations began to intensify their exploitation of marine resources through increased investment in net making. Newly available cotton could supply an existing but growing need far more efficiently than wild bast fiber plants. Moreover, as demand increased through population growth, cotton cultivation could meet and in turn underwrite further demand, in a way that a gathered wild resource never could. Cotton, then, did not merely offer relatively *minor* improvements in the technical characteristics of fiber for fishing, but rather, by hugely increasing the efficiency of *production*, it instigated a whole new world of fiber technology options. We turn next to considering how, as in other complex models (Bettinger *et al*. 2006), the socio-economic implications of such a change in raw materials here laid the Maritime Foundations of Andean Civilization.

### The Socio-economic Implications of Cotton Nets

Social inequalities in wealth, power, and status are commonly seen as born of the transition from hunter and gathering, to agriculture. Farming is, by definition, a *delayed-return* system since yields on crops or animals are only realized months or even years after labor, with implications for social relationships. Woodburn’s (1982) classic formulation on egalitarian societies, however, seeks the origins of social inequality far earlier in the human trajectory, by distinguishing the social implications of delayed-return systems in certain hunting and gathering societies, from those with immediate-return systems. Changes in the degree of such delayed-returns are evident in the archaeological record at La Yerba and indeed elsewhere all along the coast of Peru towards the end of the Middle Preceramic Period.

At La Yerba II, subsistence evidence suggests a regime whereby people obtained a direct and immediate return from their hunting and gathering labor (*cf.* “foragers” *sensu* Binford 1980). Around a millennium later, the subsistence at the settled village of La Yerba III, still dominated by hunted and gathered marine resources (*cf.* “collectors” *sensu* Binford 1980), now included important delayed-return economic features, not least some horticulture, and possibly domesticated animals. Here, fishing nets made of plant bast fibers were products of enormous labor, and were therefore “valuable technical facilities” (Woodburn 1982, 432), used and maintained over months, or indeed years. Rights over the yield of these valued assets would have been enjoyed in respect of the corporate labor invested in their making, or the cooperative venture of using them for fishing. Like the gathering of mollusks such as surf clams, net fishing is an activity in which almost all members of a community can participate, either directly or indirectly, for instance by mending nets (Norr 1975). Since fishing is variably productive through the year, levels of co-operation necessarily vary. In seasons of abundance, equipment might be pooled to deploy many nets or join nets together. Net fishing is therefore typical of a delayed-return social system entailing ordered, binding “commitments and dependencies between people” (Woodburn 1982, 433).

Increasing food returns at this time depended principally on technological improvements in fishing net production (Moseley 1975, 2005). By the Late Preceramic period, there is good evidence for greater numbers of much larger seine nets (*e.g.*, Bird *et al*. 1985, 225; Moseley and Feldman 1988). Net fishing benefits from economies of scale. Doubling the length of a beach seine net, for instance, encompasses four times the area of water. Larger nets, in turn, motivate increased social co-ordination because they require more people both to produce and to operate. Several families within a village, or even entire villages, may share in the manufacture and operation of larger nets, and the returns they yield. Larger nets would, thereby, promote wider economic links beyond the family unit and, in due course, beyond the fishing community.

Moreover, not only do larger beach seine nets take many people to lay out and land their catch, ethnographically at least (Norr 1975), their ownership tends to be become restricted to those individuals who organize their maintenance, taking a larger proportion of their catch in return and to support the making of new nets. These “net owners” often come to hold elevated prestige among their fishing communities, and it is often the ambition of others to become net owners. Thus, do larger nets foster both an unequal division of labor and increasing social hierarchy?

As we have seen, intensified marine resource exploitation at La Yerba III is reflected in increasingly sophisticated netting fabric structures, the appearance of net needles, and increases in the relative abundance of small schooling fish (including anchovy), alongside much greater, sedentary populations compared to those of La Yerba II. Indeed, certain minimum population sizes may be required to sustain and propagate the imitation of particularly skillful activities such as net making, otherwise vulnerable to imperfect inference (Henrich 2004). Such an intensification using gathered wild plant bast fibers would inevitably become circumscribed by raw material supply. The social implications of delayed yields on labor would therefore only achieve ultimate expression through the advent of cotton agriculture, in a society *pre-adapted* by its sophisticated bast fiber net technology, sedentism, and incipient horticulture of other crops.

By making larger nets cheaper to make and repair for those reasons of production discussed above, the arrival of the new raw material of cotton hugely augmented the potential to intensify marine resource exploitation, not least by increased access to the vast anchovy stocks in Peruvian seas that could be dried and stored into a protein surplus, just as envisaged in the MFAC hypothesis (Moseley 1975, 1992, 2005; Moseley and Feldman 1988).

In turn, the production of cotton fiber through cultivation entailed new ordered, differentiated, jurally defined relationships through which goods and services could be transmitted (*cf.* Woodburn 1982). Securing yields from labor, or managing assets such as fishing nets or cotton fields, increased degrees of commitment and dependency between villagers. Cotton farmers, for instance, might pool labor at certain points in the agricultural cycle such as harvesting, or for the protection of cotton crops. Larger social groups would, in turn, share rights to the land and yields from fiber.

In its latest formulation, Moseley (2005) adopts into the MFAC hypothesis Carneiro’s (1970) ideas for how competition for control of a circumscribed resource—here, land available for floodplain farming on an arid coast—could have provoked the emergence of hierarchical organization and civilization. We argue that by the Late Preceramic, a socio-economic interdependence between cotton agriculture and the co-ordination of nets made from that cotton had long since been sown in ground prepared through the much earlier inaugurations of sedentism, incipient horticulture, and the intensified exploitation of marine resources through net technologies in Middle Preceramic sites such as La Yerba.

In certain valleys of the north and central Peruvian coast, this integration of cotton farming and fishing forged those older complexities into a new agro-fishing techno-complex characterized by an intensification of social stratification and the emergence of monumental-scale sites at locations where cotton production could be concentrated on sufficiently large areas of agricultural land close to maritime resources, such as Huaca Prieta. Though there was increased reliance on food production entailing a rich suite of crops (Pearsall 2008, 116) throughout the Preceramic, human subsistence on the coast was still largely based on marine foods.

In sum, the adoption of cotton in north-central Peru by coastal societies for whom fiber production had immemorial and paramount importance for their fishing economies triggered an enormous increase in capacity to satisfy existing, perhaps even urgent, needs for a controllable, easily processed supply of fiber. Cotton thereby transformed society by driving new forms of fishing technology with greatly increased economies of scale. Larger nets fomented social complexity. Just as discussed by Métraux (1959), a *new* raw material supply for an *existing* technology precipitated revolutionary socio-economic change. Given these arguments, when cotton was adopted where, along the Peruvian coast, assumes particular significance. Fortunately, almost unique preservation conditions for an organic archaeological record in this littoral’s extreme aridity open up at least the possibility of tracing changes in plant fiber technology over millennia here.

### Moving South: Tracing a Cotton Revolution?

The earliest evidence for the use of *G. barbadense* cotton all comes from the north coast of Peru and Ecuador: whence the plant is thereby presumed to have been first cultivated (see above and Piperno 2011). Raw cotton remains associated with human settlement have been directly dated to ca. 5500 Cal bp at Real Alto, Ecuador (Damp and Pearsall 1994); ca. 6100 Cal bp at CA-09-71, Nanchoc Valley, north coast Peru (Dillehay 2011, 313); and dated by association to 7800 Cal bp CA-09-77, Nanchoc Valley (Rossen 2011, 187). Moreover, cotton artifacts are reported from Huaca Prieta, Chicama Valley, north coast Peru (Dillehay *et al*. 2012). Long associated with the Late Preceramic Period (Bird *et al*. 1985), earlier components of this site have recently been investigated yielding cotton artifacts, including yarn directly dated to 6700 Cal bp (Dillehay *et al*. 2012, 56), and textiles, dated by association to ca. 6100 Cal bp (Splitstoser *et al*. 2016). These precocious associations between cotton and the earliest suggestions of planned monumentality on the coast of Peru at Huaca Prieta (Dillehay *et al*. 2012) are striking, precisely because further south, where increasing aridity preserves an ever-older organic record, it was not until the Late Preceramic beginning around 5000 Cal bp that cotton became, as Pearsall (2008, 116) summarizes, “widespread and abundant, reflecting a focus in local economy.” In a review of the archaeobotanical evidence from the coast of Peru, Pearsall (2008, Table 7.1) notes no cotton remains for the Middle Preceramic, whereas by the subsequent Late Preceramic (or ‘Cotton Preceramic), cotton is reported as present or abundant in every reported site (see also Cohen 1978; Engel 1960, 1963b, 1987 ^5^; Moseley 1975, 1992; Moseley and Feldman 1988; Splitstoser *et al*. 2016). Moreover, as noted above, there are powerful technical reasons to suppose that the manipulation of cotton fiber into yarn *required* the innovation of spinning: the oldest evidence of which—spindle whorls—also date to the Late Preceramic, for instance at the site of El Paraíso near Lima (Moseley 1975).

## Conclusion

The Maritime Foundations of Andean Civilization were laid by innovations in plant fiber technologies. During the Middle Preceramic, the production of bast fibers twine and cordage for nets and lines was a fundamental component of the exploitation of marine resources. Fiber production must have taken up considerable time and effort—more so perhaps than any other single technological activity. Fishing nets were the products of enormous labor, used and maintained over months, or even years. Rights over these “valuable technical facilities” and their yield formed a delayed-return social system entailing ordered, binding “commitments and dependencies between people” (Woodburn 1982, 433), thereby driving social complexity and the division of labor.

By the final millennium of the Middle Preceramic, settled villages, such as La Yerba III, had been established all along the arid littoral of Peru at those places where adequate fresh water supplies were to be found. While their occupants still derived most of their subsistence from hunting and gathering, they had long been on a path of increasing social complexity. At La Yerba, this is evinced by more extensive trade or exchange networks, structured mortuary deposition connoting territoriality, increased hints of the importance of ritual, and an intensification of marine resource exploitation through increasingly sophisticated bast fiber net making: technologies that could be successfully promulgated within increasing populations. Intensification in productive systems is widely recognized as a route to social complexity (Morrison 1994), not least in the MFAC hypothesis (Moseley 1992, 2005; Moseley and Feldman 1988). Moreover, the exploitation of a broadening spectrum of resources evident at La Yerba III also entailed some floodplain horticulture. These were people who already cultivated gourds, *Phaseolus*, and *Canavalia* beans, and for whom plant fiber production had immemorial and paramount importance for their fishing economies. They were, therefore, pre-adapted to a Cotton Revolution.

Yet along the Pacific littoral south of Lima, marine resources did not, it seems, foment monumental civilization as envisaged by the MFAC hypothesis, for reasons that are instructive for the hypothesis itself. For this was *not* to any relative differences in the richness of marine resources, but rather to relative differences in the proximity of those *terrestrial* resources that determined society’s capacity to intensify exploitation of those maritime resources. The corollary of intensifying marine resource exploitation through increased specialization in fishing was, counter-intuitively, an increased demand for farmland: limited on the south coast by a geomorphology that separates extensive river floodplains from the sea, and perhaps also by river hydrologies adversely impacted by ENSO variance at this time.

Indeed, there are indications that here circumscribed gathered wild resources such as bast fiber plants had long been under pressure on the estuaries of south coast from increasing populations, so that settlement began to move upstream to sites such as at Pernil Alto, alongside increasing horticulture of food crops. In these circumstances, the nutritional requirements of these societies could perhaps be more easily and reliably met by planting beans as a protein-rich staple, and, in due course, by keeping guinea pigs.

When it arrived on the south coast, cotton was quickly integrated into a diversified, less significantly maritime, economy entailing wild and domesticated plants and animals. Following the abandonment of nearshore settlement on the south coast at the end of the Middle Preceramic after ca. 5000 bp, fishing and marine resources remained part of, and yet not essential to, the subsistence economy. The south coast became, as Kroeber (1944, 24) puts it, “coastal but not maritime” (see Carmichael *et al*. 2014).

While at first sight, therefore, the south coast archaeological record might appear to contradict the MFAC hypothesis, we argue that it calls rather for a reassessment of the role plant fibers played in the net-making technologies that allowed societies to exploit these rich maritime resources. In particular, we draw attention to the revolutionary social change precipitated by the change from fibers supplied from plants *gathered* from the local wild vegetation, to those produced from a *cultivated* crop. The effects of planting the cultivated crop of cotton into an already unfolding scenario of sedentism, intensifying marine resource subsistence and incipient agriculture, were socio-economically explosive. On the north and central coasts where broad alluvial deltas and wide ocean frontages granted simultaneous access to both rich marine and unfolding agricultural resources, cotton allowed larger nets to be manufactured and maintained far more easily, and thereby opened up the enormous potential of the ocean, particularly the prolifically abundant anchovy, to supporting ever-greater populations (Moseley and Feldman 1988; Haas and Creamer 2006). Net fishing benefits from economies of scale. Larger nets require more people to produce and operate, motivating social co-ordination and wider economic links. Thus was emerging social complexity among the coastal hunter-gatherers off the coast of Peru driven by the technological aspects of plant fiber production: firstly by the delayed-return social systems necessary to manufacture and maintain fishing nets and, in due course, through increased social stratification, division of labor, and property entailed by larger nets. Thereby refined, the Maritime Foundations of Andean Civilization hypothesis duly emerges more persuasive than ever.

In the Old World, the transition from bronze to iron entailed many factors, not least including the ability to control reducing conditions in smelting, and yet perhaps its greatest impacts came about through the change to a far more abundant and widely available source of raw materials in the form of iron oxide deposits. Civilization in Peru was likewise shaped by a social revolution precipitated by a change, to a *new*, far more abundant and reliable source of raw material—cultivated cotton—for application to an *existing* net-making technology. This Cotton Revolution, along with the domestication of camelids, eventually ushered in what Conklin (1996, 322) aptly calls an “Age of Textiles,” in which “the technology of fabric construction [became] by far the most widespread and complex material-manipulating system of Andean society.”

## Footnotes

1 Designated “L-1” by Cook (1994), it has also been called “Casavilca” (*e.g.*, Lanning 1967), after the historian who first reported the site after a visit in 1937 (Casavilca 1958), and “Morro La Gringa” (Carmichael 1991). Here, we follow Engel (1991) to avoid further proliferation of nomenclature and since he previously investigated these sites in most detail.

2 These data from La Yerba III represent very early evidence for lima beans, directly radiocarbon dated (OxA-32,290) to 6270–5996 Cal bp, and jack beans and guinea pigs, dated by association to 6281–6006 Cal bp. Quilter (1992, 114) notes that there is no previous evidence of domesticated guinea pig during the Preceramic , which might have provided a significant source of animal protein. Since wild guinea pig are seen in the riparian oases of the lower Ica Valley today, it remains unclear, however, whether these La Yerba III remains are evidence of animal husbandry, or of hunting.

3 The beginning of the Late Preceramic (or Preceramic VI or Late Archaic) Period is usually given as 4450 bp uncalibrated (Quilter 1991a, 1991b). Calibrated using ShCal13 (Hogg *et al*. 2013), this is about 5000 Cal bp. All dates cited herein are calibrated unless otherwise specified.

4 Information on modern fishing practices was gathered from itinerant fishermen at the mouth of the Río Ica, in particular from Snr. Reynaldo Lobato, who has earned his living by fishing here for most of his 58 years.

5 Engel’s diverse publications are sometimes incoherent on certain details: a case in point being information on cotton at Middle Preceramic sites, which otherwise share many features with La Yerba III. For instance, his discussion of the Chilca I village argues, on the one hand, that there is no evidence that cotton was used for the making of yarns (Engel 1987, 53), and on the other, that cotton was recovered from the deepest parts of its stratigraphy (Engel 1987, 43). For the south coast, Engel (1960, 1963b) states explicitly in early publications immediately after his 1950s fieldwork that cotton was *not* associated with the earliest sedentary agricultural societies of the south coast, at those sites on the estuary of the Río Grande de Nazca and at Paracas. Yet, in later syntheses (“what I now notice, twenty years later” Engel 1981, 21), he makes general references to cotton at both Santa Ana at the estuary of the Río Grande de Nazca, and at Village 514 on Pampa Santo Domingo, Paracas. Indeed, he renames the latter “Algodón y Jícama” (Engel 1991, 69–83), from which seven radiocarbon dates are published, six of which are Middle Preceramic, once calibrated (using Hogg *et al*. 2013, see Fig. 2). The identification of cotton here rests upon an assertion that it “was made by the Smithsonian Institution, Washington, who carried out the analysis of the garments of 12 individual interred at this site and the wefts of their basketry wrappings” (Nicho *et al*. 2016, 31, *our translation*). None of these later general statements specify that cotton plant remains were recovered and those few fabrics from these sites that have been illustrated and analyzed do not seem to be of cotton (Nicho *et al*. 2016). Pending further evidence or analysis, such contradictions and uncertainties lead us to prefer to rely on those earlier interpretations of Engel's, by which he defined his 'Cotton Preceramic' (Moseley 1992: 9), and conservative reviews of the archaeobotanical evidence (*e.g.*, Pearsall 2008, 116).
